# When Do HIV-Infected Women Disclose Their HIV Status to Their Male Partner and Why? A Study in a PMTCT Programme, Abidjan

**DOI:** 10.1371/journal.pmed.0040342

**Published:** 2007-12-01

**Authors:** Hermann Brou, Gérard Djohan, Renaud Becquet, Gérard Allou, Didier K Ekouevi, Ida Viho, Valériane Leroy, Annabel Desgrées-du-Loû, ANRS 1201/1202/1253 Ditrame Plus Study Group

**Affiliations:** 1 Laboratoire Population Environnement Développement, Institut de recherche pour le développement, Nogent-sur-Marne, France; 2 Projet ANRS 1253 Ditrame Plus, Ecole Nationale Supérieure de Statistiques et d'Economie Appliquée, Abidjan, Côte d'Ivoire; 3 INSERM, Unité 593, Bordeaux, France; 4 Institut de Santé Publique Epidémiologie et Développement, Université Victor Segalen Bordeaux 2, Bordeaux, France; 5 Projet ANRS 1201/1202 Ditrame Plus, Programme PAC-CI, Centre Hospitalier Universitaire de Treichville, Abidjan, Côte d'Ivoire; National Institute of Child Health and Human Development, United States of America

## Abstract

**Background:**

In Africa, women tested for HIV during antenatal care are counselled to share with their partner their HIV test result and to encourage partners to undertake HIV testing. We investigate, among women tested for HIV within a prevention of mother-to-child transmission of HIV (PMTCT) programme, the key moments for disclosure of their own HIV status to their partner and the impact on partner HIV testing.

**Methods and Findings:**

Within the Ditrame Plus PMTCT project in Abidjan, 546 HIV-positive and 393 HIV-negative women were tested during pregnancy and followed-up for two years after delivery. Circumstances, frequency, and determinants of disclosure to the male partner were estimated according to HIV status. The determinants of partner HIV testing were identified according to women's HIV status. During the two-year follow-up, disclosure to the partner was reported by 96.7% of the HIV-negative women, compared to 46.2% of HIV-positive women (χ^2^ = 265.2, degrees of freedom [df] = 1, *p* < 0.001). Among HIV-infected women, privileged circumstances for disclosure were just before delivery, during early weaning (at 4 mo to prevent HIV postnatal transmission), or upon resumption of sexual activity. Formula feeding by HIV-infected women increased the probability of disclosure (adjusted odds ratio 1.54, 95% confidence interval 1.04–2.27, Wald test = 4.649, df = 1, *p* = 0.031), whereas household factors such as having a co-spouse or living with family reduced the probability of disclosure. The proportion of male partners tested for HIV was 23.1% among HIV-positive women and 14.8% among HIV-negative women (χ^2^ = 10.04, df = 1, *p* = 0.002). Partners of HIV-positive women who were informed of their wife's HIV status were more likely to undertake HIV testing than those not informed (37.7% versus 10.5%, χ^2^ = 56.36, df = 1, p < 0.001).

**Conclusions:**

In PMTCT programmes, specific psychosocial counselling and support should be provided to women during the key moments of disclosure of HIV status to their partners (end of pregnancy, weaning, and resumption of sexual activity). This support could contribute to improving women's adherence to the advice given to prevent postnatal and sexual HIV transmission.

## Abstract 2

In a mother-to-child HIV prevention program in Côte d'Ivoire, Annabel Desgrées-du-Loû and colleagues identify three junctures at which women tend to disclose their HIV status to partners.

## Editors' Summary

**Background.:**

Since the first reported case of AIDS (acquired immunodeficiency syndrome) in 1981, the number of people infected with the human immunodeficiency virus (HIV), which causes AIDS, has risen steadily. By the end of 2006, nearly 40 million people were infected, 25 million of them in sub-Saharan Africa. HIV is most often spread by having unprotected sex with an infected partner. In Africa, most sexual transmission of HIV is between partners in stable relationships—many such couples do not adopt measures that prevent viral transmission, such as knowing the HIV status of both partners and using condoms if one partner is HIV-positive. HIV can also pass from a mother to her baby during pregnancy, labor, or delivery, or through breastfeeding. Mother-to-child transmission (MTCT) of HIV can be reduced by giving anti-HIV drugs to the mother during pregnancy and labor and to her newborn baby, and by avoiding breastfeeding or weaning the baby early.

**Why Was This Study Done?:**

Many African countries have programs for prevention of MTCT (PMTCT) that offer pregnant women prenatal HIV counseling and testing. As a result, women are often the first member of a stable relationship to know their HIV status. PMTCT programs advise women to disclose their HIV test result to their partner and to encourage him to have an HIV test. But for many women, particularly those who are HIV-positive, talking to their partner about HIV/AIDS is hard because of fears of rejection (which could mean loss of housing and food) or accusations of infidelity. Knowing more about when women disclose their HIV status and what makes them decide to do so would help the people running PMTCT programs to support women during the difficult process of disclosure. In this study, the researchers have investigated when and why women participating in a PMTCT research project in Abidjan (Côte d'Ivoire) told their partner about their HIV status and the impact this disclosure had on their partner's uptake of HIV testing.

**What Did the Researchers Do and Find?:**

At regular follow-up visits, the researchers asked women in the Abidjan PMTCT project whether they had told their partners their HIV status and whether they were breast-feeding or had resumed sexual activity. Nearly all the women who tested negative for HIV, but slightly fewer than half of the HIV-positive (infected) women had told their partner about their HIV status by two years after childbirth. Two-thirds of the HIV-positive women who disclosed their status did so before delivery. Other key times for disclosure were at early weaning (4 months after birth) for women who breast-fed their babies, and when sexual activity resumed. HIV-positive women who bottle fed their babies from birth were more likely to tell their partners of their status than women who breast-fed. Factors that prevented women disclosing their HIV status included living in a polygamous relationship or living separately from their partners. Finally, the researchers report that the partners of HIV-positive women who disclosed their HIV status were about three times more likely to take an HIV test than the partners of HIV-positive women who did not disclose.

**What Do These Findings Mean?:**

These findings identify three key times when women who have had an HIV test during pregnancy are likely to disclose their HIV status to their partner. The main one is before delivery and relates, in part, to how the mother plans to feed her baby. To bottle feed in Abidjan, women need considerable support from their partners and this may be the impetus for disclosing their HIV status. Disclosure at early weaning may reflect the woman's need to enlist her partner's support for this unusual decision—the normal time for weaning in Abidjan is 17 months. Finally, disclosure when sexual activity resumes may be necessary so that the woman can explain why she wants to use condoms. Although these findings need confirmation in other settings, targeting counseling and support within PMTCT programs to these key moments might help HIV-positive women to tell their partners about their status. This, hopefully, would help to reduce sexual transmission of HIV within stable relationships in sub-Saharan Africa.

**Additional Information.:**

Please access these Web sites via the online version of this summary at http://dx.doi.org/10.1371/journal.pmed.0040342.

- Information is available from the US National Institute of Allergy and Infectious Diseases on HIV infection and AIDS and on HIV infection in women
- HIV InSite has comprehensive information on all aspects of HIV/AIDS
- Women Children and HIV provides extensive information on prevention of mother-to-child transmission of HIV in developing countries
- Information is available from Avert, an international AIDS charity, on HIV and AIDS in Africa and on HIV and AIDS prevention
- AIDSinfo, a service of the US Department of Health and Human Services provideshealth information for HIV-positive pregnant women (in English and Spanish)

## Introduction

At the end of 2006, 63% of all people livingwith HIV/AIDS lived in sub-Saharan Africa [1]. Programmatic strategies for the prevention of sexual transmission of HIV need urgent development, assessment, and scale-up [2]. In African countries confronted with an HIV/AIDS pandemic, most cases of sexual transmission of HIV occur within stable relationships. In sub-Saharan Africa, Prevention within the couple is therefore of primary importance. In 2006, 59% of HIV-infected adults were women [1], and most of them had contracted HIV through sexual transmission from their stable partner [3].

Most studies available on sexual relations within the couple in sub-Saharan Africa have shown lack of prevention of sexual transmission of HIV within stable relationships [4,5]. The prevention of sexual transmission of HIV within the couple involves HIV testing for each member and the systematic use of condoms if one of the members is HIV-positive or until both members have been tested HIV-negative and have adopted safe sex practices. Research studies exploring how the risk of sexual transmission of HIV infection is managed within couples in sub-Saharan Africa show that these simple principles are unfortunately rarely implemented [6–8]. Sexual relations with the regular partner are rarely protected, because they are perceived as risk-free [4,5]. Nevertheless, in populations with a high prevalence of HIV infection, those who engage in conjugal sexual relations are at risk of infection. HIV testing of each partner and conjugal exchange of information on serostatus remain the only way to evaluate the risk of HIV transmission in conjugal sexual relations. Nonetheless, HIV testing has remained infrequent in Africa [9].

With the implementation of prevention of mother-to-child transmission of HIV (PMTCT) programmes in African countries, prenatal HIV counselling and testing is offered to many pregnant women. Hence these women are often the first to be HIV tested within couples [10]. These women are then counselled to share with their partner their own HIV test result, and they become responsible for encouraging their partner to undertake HIV testing. But the dialogue on sexual activity or HIV/AIDS within the couple is not easy, especially when women discover that they are HIV-infected [11–13]. Available studies have documented women's experience of disclosure to their partner and reported the barriers to disclosure, such as women's fears related to stigmatisation, family rejection, breach of confidentiality, or accusations of infidelity [14]. These studies, however, did not explore the dynamic of the woman's decision when she informed her partner of her HIV status. Better understanding of the circumstances and events leading to women's disclosure to their partner is required in order to better support them in this process. In this paper, we investigated which women who accepted HIV testing within a PMTCT programme reported their HIV status to their partner, and when they did so between HIV testing in pregnancy and 24 months after delivery. We also examined whether or not telling the partner had led to HIV testing of the partner.

## Methods

### Ditrame Plus Research Programme

The ANRS 1201/1202/1253 Ditrame Plus programme was the PMTCT research effort implemented in Abidjan, Côte d'Ivoire in March 2001 [15–17]. HIV testing was systematically offered at the first antenatal consultation to all pregnant women aged 18 y or over who attended one of the seven antenatal clinics located in two poor, densely populated districts of Abidjan. After signing an informed consent form, women were regularly followed up for 2 y after delivery, every 3 mo during the first year and every 6 mo during the second year. The Ditrame Plus study was granted ethical permission in Côte d'Ivoire from the ethical committee of the National AIDS Control Programme, and in France from the institutional review board of the French Agence Nationale de Recherches sur le Sida (ANRS).

Consenting HIV-positive women were systematically invited to be included in a cohort offering PMTCT interventions (fully described elsewhere [16,17]): short-course peripartum antiretroviral regimens and exclusive formula feeding from birth until 9 mo postpartum or exclusive breast-feeding with early cessation at 4 mo. A subgroup of HIV-negative pregnant women were also included and followed up in another cohort offering HIV counselling, contraception access, and access to care. During pre- and post-test counselling and postpartum follow-up, all women were informed regarding sexually transmitted infections, including HIV/AIDS, and the use of condoms. After delivery, they were also offered postnatal contraception for 1 mo after delivery and free provision of contraceptives including condoms.

HIV-negative and HIV-positive women attended different clinics. During each follow-up visit, standardised questionnaires were administered to all women to document the disclosure of HIV status to the partner, the resumption of sexual activity, and sociodemographic characteristics. The same standardised questionnaire was used in the two prospective cohorts of HIV-positive and HIV-negative women for comparative analysis.

### Population

From March 2001 to June 2003, 980 pregnant women who were tested for HIV during antenatal consultation and who had delivered were included within the Ditrame Plus programme. The average age of gestation of HIV-infected pregnant women was 36 wk [range 26–43] at enrolment. Excluded from this analysis were 23 (2.3%) women lost to follow-up before the visit scheduled at 1 mo after delivery and 18 (1.8%) women who remained without any partner during the follow-up period. A total of 939 women, of whom 546 were HIV-positive and 393 HIV-negative, were included for this analysis. Remaining in the study through the 18-mo postpartum appointment were 90% of HIV-negative women and 85% of HIV-positive women (χ^2^ = 6.603, degrees of freedom [df] = 1, *p* = 0.010).

### Disclosure of HIV Status to Partner

**Figure 1:**
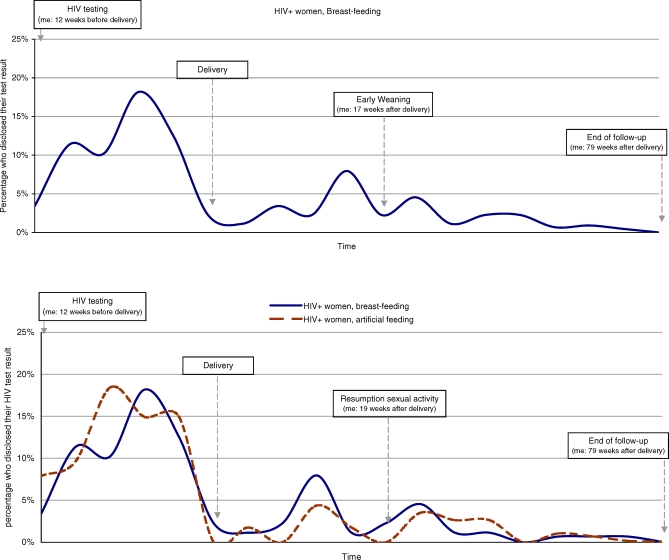
Distribution of the Moment when HIV-Infected Women Disclosed Their Status to Their Male Partner between HIV Testing and the End of Follow-Up (Ditrame Plus ANRS 1201-1202-1253, Abidjan, 2001–2005) (A) In relation to delivery and weaning. (B) In relation to delivery and resumption of sexual activity. See Text S1 for details on the construction of the curves. me, median.

We analysed the timing of women's disclosure of their HIV status to their partner. As the exact date was not known, we estimated the disclosure date as the mid-period between the date of the previous follow-up visit and the date of the visit when the woman reported having disclosed her status. We then compared this period to specific events occurring between prenatal HIV testing and at the time of resuming sexual activity. Specific questions were asked at each visit on the date of resumption of sexual activity and the date of cessation of breast-feeding. We analysed the distribution of the disclosure moment between HIV testing and the end of the follow-up in relation to delivery, resumption of sexual activity, and weaning by women who chose to breast-feed. Figure 1 illustrates the distribution of the time of disclosure, between HIV testing and the end of the follow-up, in relation to delivery, resumption of sexual activities, and weaning for breast-feeding women. (See Text S1 for details of how the curve was constructed.)

### Partner's HIV Testing

Only partners HIV tested in the 2 y prior to the start of the Ditrame Plus programme or after the start of the programme, and whose HIV results were known, were taken into account in this analysis (15 partners tested for HIV before March 1999 were therefore excluded). The proportion of partners tested for HIV was described according to the sociodemographic characteristics of the women, partner, and couple.

### Statistical Tools

Statistical analyses were first performed on all women followed up and then within each cohort of women according to their HIV status. Univariate analyses comprised: variables related to the woman (i.e., age, religion, education level, remunerated activity, parity, existence of a co-spouse, type of habitat, number of cohabiting family members, HIV status, and clinical AIDS stage for HIV-infected women, according to the WHO Staging System of HIV Infection and Disease); variables related to the partner (i.e., age, education level, and HIV status); and variables related to the infant followed up within the project (i.e., infant feeding practice implemented at birth and child survival). Group comparisons used nonparametric Mann–Whitney U test for quantitative variables, and χ^2^ or Fisher exact tests for qualitative variables. Multivariate logistic regressions were performed and included all variables. All statistical analyses were conducted with SPSS for Windows (version 12.0).

## Results

### Characteristics

Table 1 describes the sociodemographic characteristics of both cohorts of women and their partner. HIV-positive women were slightly older than their HIV-negative counterparts, and more often lived within a polygamous household (21.8% versus 12.5%, χ^2^ = 13.53, df = 1, *p* < 0.001). Male partners were on average more educated and older than their wives. All HIV-negative women who delivered live infants practiced breast-feeding. Among the 546 HIV-positive women in the study, 243 (44.5%) breast-fed their infant with early cessation at 17 wk in median (interquartile range [IQR 13–32]), 283 (51.8%) practiced formula feeding, and for 18 (3.7%) the information was not reported. Among these women, 88.9% of HIV-infected women who practiced breast-feeding and 92.5% who practiced formula feeding complied with the choice expressed prior to delivery (χ^2^ = 2.147, df = 1, *p* = 0.143).

**Table 1:**
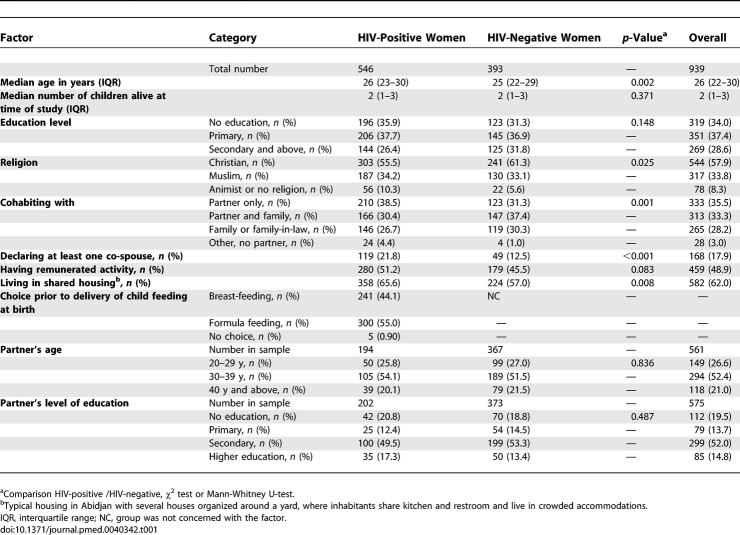
Sociodemographic Characteristics of Women at Enrolment According to Their HIV Status (Ditrame Plus ANRS 1201-1202-1253, Abidjan, 2001–2005)

### Women's Disclosure of Their HIV Status to Their Partner

Most of the HIV-negative women (96.7%) disclosed their HIV result to their partner, compared to 46.2% of HIV-positive women (χ^2^ = 265.2, df = 1, *p* < 0.001). Among HIV-infected women who disclosed their HIV status, 82.1% declared that their partner had a “positive” reaction, i.e., was understanding and provided moral support. Among the women declaring “negative” reactions from their partner after disclosure, 10 (4%) were blamed for not discussing with him prior to HIV testing, one (0.4%) experienced violence, six (2.4%) ended their relationship with their partner, and five (2%) declared their partner did not believe their wife's positive test result.

As Table 2 indicates, HIV-infected women were less likely to disclose their HIV status when they lived with their own family but without their partner than when they lived with their partner only (adjusted odds ratio [OR] = 0.29, 95% confidence interval [CI] 0.17–0.50, Wald test = 20.68, df = 1; *p* < 0.001) and when they had a co-spouse, versus being the only wife (adjusted OR = 0.51, 95% CI 0.31–0.83, Wald test = 7.19, df = 1, *p* = 0.007). The probability of disclosing to the partner was higher for HIV-infected women who chose formula feeding than for those initiating breast-feeding after birth (OR = 1.54, 95% CI 1.04–2.27, Wald test = 4.649, df = 1; *p* = 0.031). No significant correlation was found between disclosure and whether or not the woman was engaged in remunerated activity.

**Table 2:**
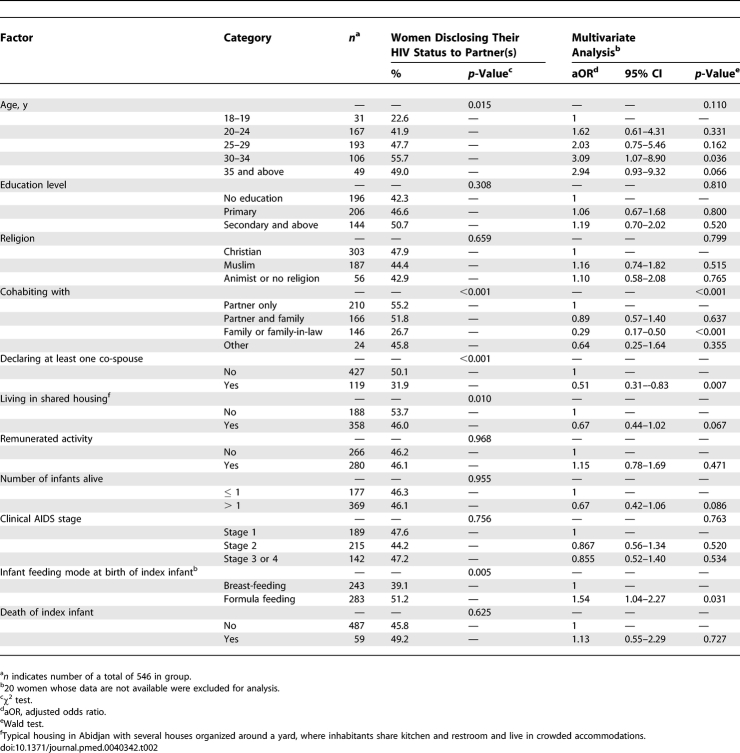
Determinants of Women's HIV Status Disclosure to Partners, among HIV-Infected Women: Univariate Analysis and Multivariate Logistic Regression (Ditrame Plus ANRS 1201-1202-1253, Abidjan, 2001–2005)

The majority of HIV-infected women disclosed their HIV status to their partner before delivery. Among breastfeeding HIV-infected women who disclosed their HIV status to their partner, 55.7% disclosed before delivery, 17% between delivery and the resumption of sexual activity, and 19% between delivery and weaning. Among HIV-infected women who did not breast-feed, 65.8% disclosed before delivery and 8% disclosed between delivery and resumption of sexual activity.

Disclosure before delivery was related to child feeding choice: among women who disclosed their HIV status before delivery, 34.6% decided to breast-feed and 64.8% decided not to breast-feed prior to delivery (χ^2^ = 12.35, df = 1, *p* < 0.001).

Among the women who disclosed after delivery, we observed peaks of disclosure just around the period of weaning and around the resumption of sexual activity (Figure 1).

### Partners' HIV Testing

Overall, 184 (19.6%) partners were tested for HIV. Partners of HIV-positive women were more likely to be tested than partners of HIV-negative women (23.1% versus 14.8%, χ^2^ = 10.04, df = 1, *p* = 0.002). Among the 184 couples HIV tested, 54 (29.4%) were seroconcordant HIV-positive, 56 (30.4%) were seroconcordant HIV-negative and 74 (40.2%) were serodiscordant couples. In the serodiscordant couples, two women were HIV-negative and 72 women were HIV-positive.

For partners of HIV-negative women, demographic variables such as education and marital status (monogamous or polygamous) were not correlated with HIV testing (Table 3). The only variable significantly associated with partner HIV testing was previous HIV testing of the partner (44.4% versus 14.1%, χ^2^ = 6.452, df = 1, *p* = 0.011). For partners of HIV-positive women, on the other hand, partners were more likely to be tested if they were educated (46.3% versus 16.7%, χ^2^ = 12.12, df = 1, *p* < 0.001), were informed of their wife's infection (37.7% versus 10.5%, χ^2^ = 56.36, df = 1, *p* < 0.001), in a monogamous couple (27.6% versus 6.7%, χ^2^ = 22.93, df = 1, *p* < 0.001), and had previous HIV testing experience (100% versus 22%, χ^2^ = 20.22, df = 1, *p* < 0.001).

**Table 3:**
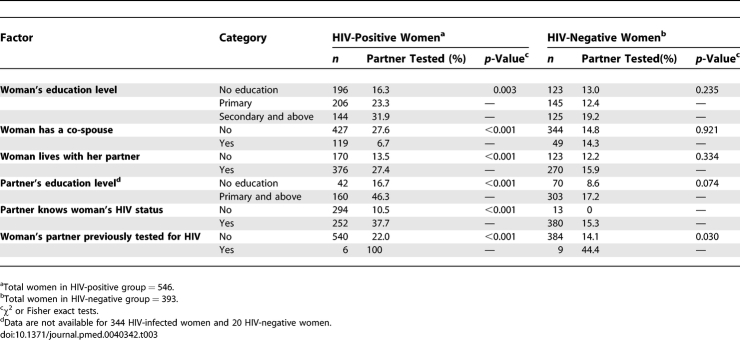
Proportions of Male Partners Tested for HIV According to Women's HIV Status (Ditrame Plus ANRS 1201-1202-1253, Abidjan, 2001–2005)

## Discussion

In this study, almost all (96.7%) women who had been informed of their HIV-negative status notified their partner. Among HIV-infected women, less than half (46.2%) had disclosed to their partner at the end of the follow-up period. We have also highlighted the existence of three privileged moments for HIV-infected women's disclosure to their partner: before delivery, upon resumption of sexual activity, and around early weaning for breast-feeding women. For HIV-negative women, we had already described in a previous study that most of them informed their partner of their testing before delivery [7]. Only one in five male partners were tested for HIV. Partners informed of their wife's HIV status were more likely to undertake HIV testing, in particular when the woman tested HIV-positive.

This study was conducted within a population of women participating in a research programme proposing systematic prenatal HIV testing and counselling but not in the operational context of PMTCT services delivery. The psychosocial support of women in our study may have been stronger than in an operational context. Hence the proportions of women who disclosed their HIV status to their partner and the proportion of partners HIV tested may be higher than those obtained in an operational context. Another weakness is that we did not study the partner's reaction over a long period of time. Hence the negative reactions of partners may be underestimated. However, two studies in Tanzania and Burkina Faso had previously showed that after sharing information, most male partners proved to be understanding and the majority of the couples remained stable [18,19]. Despite these weaknesses, our prospective study design provides reliable and original information on the timing of women's disclosure of their HIV status to their partner. Our timing data were very precise before delivery (66% of disclosure), because women were seen every other week. After delivery, women were only seen every three months and data were less precise, but even in this period we had precise information on the relative position of the different events: disclosure, resumption of sexual activity, and weaning.

Our results reveal that, in spite of the continual counselling and psychosocial support provided within the Ditrame-Plus programme, the proportion of HIV-infected women who disclosed their status to their partner is comparable to that observed in the context of a previous PMTCT research programme conducted on the same sites in Abidjan and with no specific support by the sociomedical team for notifying the partner [20]. This lack of evolution over time underlines the difficulties HIV-infected women encounter in discussing their own HIV status within the couple and raises the persistent fear of social stigma associated with HIV in this context. Nevertheless, these HIV-infected women declared during qualitative interviews published elsewhere [8] that disclosing their status to their partner seemed essential to them, so the women could benefit from comfort and support to make important decisions for their infant and the couple. Most (82%) of the partners of HIV-infected women informed were understanding and provided their wife with moral support. Negative reactions of the male partner (violence, separation) existed but they were rare.

The analysis of disclosure over time showed that two-thirds of the HIV-infected women who disclosed their HIV status to their partner reported they did so before delivery. This result may be related to the fact that during pregnancy, women were asked to say how they intended to feed the child they were carrying, i.e., formula feeding from birth or exclusive breast-feeding with early cessation at four months. Two-thirds (64.8%) of women who had disclosed their HIV status before delivery opted for formula feeding, versus 50% among women who did not disclose before delivery. Indeed, in this context, where breast-feeding is widely practiced and prolonged [21], it was important for HIV-infected women opting for formula feeding to receive their partner's support. Hence they were more likely to disclose their HIV status before delivery, i.e., before implementation of the infant feeding choice. This is an important factor to take into account in the prevention of MTCT. Similarly, for women who breast-fed their child at birth, the period around weaning appeared to be a critical moment for disclosure to the partner [22]. The median duration (4 mo) of total breast-feeding among HIV-infected women in the Ditrame Plus study was considerably shorter than what was previously described in Abidjan where median duration was estimated to be around 17 mo [21]. In the Ditrame Plus programme, we observed that failure of early weaning was linked to pressure from the woman's family-in-law. [17]. Breast-feeding HIV-infected women who had not disclosed their HIV status before delivery may have chosen to do so at weaning time, in order to justify early weaning to the partner and get his support in front of the family and community. An earlier study conducted in Abidjan and Bobo-Dioulasso noted that the partner's opinion was the first obstacle to adoption of safe infant feeding practices to prevent HIV transmission through breast-feeding [23]. The implementation of alternatives to prolonged breast-feeding for PMTCT depends heavily on the conjugal and social environment of each HIV-infected woman.

Finally, a third event appeared to be essential in the disclosure process, the resumption of sexual activity after delivery. When women were informed of their HIV infection, they received counselling on preventing transmission via sexual relations. When they resume sex, proposing the use of condoms to their partner is complex and arouses suspicion if the partner is unaware of his wife's HIV status. Disclosing her HIV infection may seem necessary to avoid HIV transmission within the couple.

At these key moments for disclosure that we identified, intensified psychosocial support for women may increase the proportion of women who manage to disclose their HIV status to their partner. The women who would benefit from such support around disclosure are those who encounter difficulties in the present programme in talking with their partner, i.e., mainly the youngest women with less conjugal experience and those whose living conditions are not suitable for conjugal confidentiality (shared housing, no co-residence with the male partner, or presence of a co-spouse).

Conjugal organisation seems to be an important determinant of disclosure. Women cohabiting with their partner were more likely to share their test result, regardless of their HIV status. Similar results were observed in a Zambian study on individual and couple HIV counselling and testing [24]. Cohabitation indeed provides more space and time for discussing such sensitive issues as HIV infection. By contrast, living in polygamous households or in shared housing reduces the likelihood of women's disclosure, probably due to reduced confidentiality. It seems that women who do not live with their partner and/or who have a co-spouse are less likely to trust their partner.

Only 19.6% of male partners were tested for HIV. The programme did not offer any couple HIV counselling and testing, but free HIV counselling and testing were available to any willing partner. Three reasons may explain the small proportion of men tested for HIV [7]: the fear of discovering his HIV-positive status; the need to personally and actively request HIV testing (unlike pregnant women, who were offered HIV counselling and testing during antenatal care); and the belief that HIV status matched the wife's. This latter reason, i.e., the belief that couples cannot be serodiscordant, may explain why only 14.8% male partners of HIV-negative women were tested. A similar result was found in Tanzania: only 16% of partners informed of their wife's HIV status said they would like to go for testing [11].

Partners' HIV testing was significantly correlated to their previous HIV testing experience, in addition to others factors such as education level and sharing of woman's HIV test result. Earlier experience with HIV testing seems to diminish the fear associated with the test, so it would be valuable to multiply the occasions to be HIV tested.

In conclusion, our study suggests that the implementation of specific psychosocial counselling and support for HIV-infected women at the end of pregnancy, the period of early weaning, and the resumption of sexual activity are important to help women to disclose their HIV status to their partner. This disclosure is an important step that could contribute to improving women's adherence to the advice given to prevent postnatal and sexual HIV transmission.

## Supporting Information

Alternative Language Abstract S1 Translation of the Abstract into French (28 KB DOC)

Text S1 Appendix: Contruction of Curves in Figure 1
(27 KB DOC)

## Abbreviations

CI: confidence interval
df: degrees of freedom
OR: odds ratio
PMTCT: prevention of mother-to-child transmission of HIV
