# Supplementary material for: When Do HIV-Infected Women Disclose Their HIV Status to Their Male Partner and Why? A Study in a PMTCT Programme, Abidjan
Source: PLoS Med. 2007 Dec 1;4(12):e342. doi: 10.1371/journal.pmed.0040342 (PMC2100145; doi:10.1371/journal.pmed.0040342)
Supplement: Supplementary file 1 [file pmed.0040342.sd001.doc]

**Resumé**

**Contexte:** En Afrique, les femmes dépistées pour le VIH en consultation prénatale sont encouragées à partager avec leur partenaire masculin le résultat de ce test de dépistage et à lui conseiller de se faire dépister. Nous avons analysé dans le cadre d’un programme de prévention de la transmission mère-enfant du VIH quels étaient les moments-clés de cette information du partenaire, et son impact sur le dépistage du partenaire.

**Méthodes et résultats :** Au sein du programme de PTME Ditrame Plus à Abidjan, 546 femmes infectées par le VIH et 393 femmes séronégatives ont été dépistées au cours d’une grossesse et suivies deux ans après l’accouchement.. Nous avons mesuré, selon le séro-statut des femmes, le moment auquel elles ont informé leur partenaire de leur dépistage. Les fréquences et déterminants de cette information et du dépistage du partenaire ont été analysés. Au cours des deux ans de suivi, 96.7% des femmes séronégatives ont informé leur partenaire de leur dépistage, contre 46 .2% des femmes infectées par le VIH (χ²=265.2, df=1, p<0.001). Chez les femmes VIH+, cette information s’est faite généralement soit juste avant l’accouchement, soit au moment du sevrage précoce (à 4 mois pour éviter la transmission postnatale), soit autour de la reprise des rapports sexuels. La probabilité d’informer le partenaire était plus grande chez les femmes VIH+ qui avaient choisi l’allaitement artificiel (odds ratio ajusté : 1.54, intervalle de confiance à 95%: 1.04-2.27, Wald test=4.649, df=1, p=0.031). Elle était plus faible chez les femmes ayant une co-épouse ou vivant avec leur famille. Parmi les partenaires de femmes VIH+, 23.1% ont été dépistés contre 14.8% parmi les partenaires de femmes séronégatives (χ²=10.04, df=1, p=0.002). La probabilité d’être dépisté était plus importante si ils étaient informés du séro-status de leur femme que s’ils ne l’étaient pas (37.7% versus 10.5%, χ²=56.36, df=1, p<0.001).

**Conclusions:** Dans les programmes de PTME, les femmes devraient bénéficier d’un soutien psychosocial spécifique pour informer leur partenaire de leur séropositivité lors de trois étapes-clés : la fin de la grossesse, le sevrage du bébé et la reprise des rapports sexuels. Cela permettrait d’améliorer la mise en pratique des conseils qu’elles reçoivent pour prévenir la transmission postnatale et la transmission sexuelle du VIH.

**Mots-clés :** Dépistage VIH prénatal, Afrique, femmes, information du partenaire, dépistage VIH du partenaire
