# Supplementary material for: When Do HIV-Infected Women Disclose Their HIV Status to Their Male Partner and Why? A Study in a PMTCT Programme, Abidjan
Source: PLoS Med. 2007 Dec 1;4(12):e342. doi: 10.1371/journal.pmed.0040342 (PMC2100145; doi:10.1371/journal.pmed.0040342)
Supplement: Supplementary file 2 [file pmed.0040342.sd002.doc]

**Appendix**

**1- Construction of curves of Figure 1**

On these two curves we situated disclosure of the woman’s HIV-status to her partner during the period between HIV-testing and the end of the follow-up. During this period the disclosure moment is related to the delivery, the resumption of sexual activity and the weaning of the child for breastfeeding women, in term of anteriority/posteriority and duration. On this time scale specific to each woman, the disclosure moment is located between HIV-testing and delivery, if disclosure occurred before delivery. If it occured between delivery and the end of follow-up, it is located in regard to weaning (Fig. 1a) or resumption of sexual activity (Fig. 1b). Resulting curves show frequencies of these relative moments. This graphical construction allows to characterise the distribution of the disclosure moments in relation with three key peri- or postpartum events: the delivery, the resumption of sexual activity and the weaning of the child for breastfeeding women.

*Figure 1a*: For each woman, the follow-up is scaled according to four specific moments: **d_0_** date of announcement of the HIV-test result to the woman; **d_1_** date of delivery (or end of pregnancy); **d_2_** date of weaning; **d_f_** date of end of follow-up. The abscissa of the moment at which the woman disclosed her HIV-status to her partner (**d**) is estimated according to these moments, using a ratio **r** calculated as follows:

If disclosure before delivery:	r = (d- d_1_) / (d_1_ – d_0_)	⇒ -1 ≤ r ≤ 0.

If disclosure after delivery:	r = (d - d_1_) / (d_2_ – d_1_)	⇒ 0 ≤ r.

Hence, for each woman: d_0_ on abscissa (-1), d_1_ on abscissa (0), d_2_ on abscissa (1), the disclosure moment on abscissa (r), which can take values comprised between -1 (disclosure happened at the same time as announcement) and 90 (disclosure happened at the end of follow-up and weaning happened one week after delivery). On ordinate, we indicate the proportion of women having disclosed their status to their partners, calculated for each step of 0.2 until r = 2. After r = 2, the step is increased and the proportion of women having disclosed their status to their partners is adjusted according to the step. Then the slope was smoothed.

*Figure 1b*: same as above, with the date of resumption of sexual activity instead of the date of weaning.

**2- Members of the ANRS 1201-1202-1253 Ditrame Plus Study Group**

Medical team:

##### *Principal Investigators:* François Dabis, Valériane Leroy, Marguerite Timite-Konan, Christiane Welffens-Ekra.

##### *Coordination in Abidjan:* Laurence Bequet, Didier K. Ekouevi, Besigin Tonwe-Gold, Ida Viho.

##### *Methodology, biostatistics and data management:* Gérard Allou, Renaud Becquet, Katia Castetbon, Laurence Dequae-Merchadou, Charlotte Sakarovitch, Dominique Touchard.

##### *Clinical team:* Clarisse Amani-Bosse, Ignace Ayekoe, Gédéon Bédikou, Nacoumba Coulibaly, Christine Danel, Patricia Fassinou, Apollinaire Horo, Ruffin Likikouët, Hassan Toure.

##### *Laboratory team:* André Inwoley, François Rouet, Ramata Touré. *Psycho-social team*: Hortense Aka-Dago, Alphonse Sihé.

##### *Scientific Committee:* Stéphane Blanche, Jean-François Delfraissy, Philippe Lepage, Laurent Mandelbrot, Christine Rouzioux, Roger Salamon.

Social Science team:

Principal Investigators : Annabel Desgrées du Loû, Benjamin Zanou.

Coordination in Abidjan and quantitative survey: Hermann Brou.

Qualitative survey: Annick Tijou-Traore, Hélène Agbo.

Data Management: Gerard Djohan.

The Ditrame Plus study was granted ethical permission in Côte d’Ivoire from the ethical committee of the National AIDS Control Programme, and in France from the institutional review board of the French Agence Nationale de Recherches sur le Sida (ANRS).
